# Information standards for recording alcohol use in electronic health records: findings from a national consultation

**DOI:** 10.1186/s12911-018-0612-z

**Published:** 2018-06-07

**Authors:** Shamil Haroon, Darren Wooldridge, Jan Hoogewerf, Krishnarajah Nirantharakumar, John Williams, Lina Martino, Neeraj Bhala

**Affiliations:** 1 Institute of Applied Health Research, College of Medical and Dental Sciences, University of Birmingham, Edgbaston, Birmingham, B15 2TT UK; 2 Health Informatics Unit, Royal College of Physicians, 11 St Andrews Place, Regent’s Park, London, NW1 4LE UK; 3 Public Health Specialty Registrar, West Midlands, UK; 4 Queen Elizabeth Hospital Birmingham, Mindelsohn Way, Birmingham, B15 2TH UK

**Keywords:** Alcohol, Electronic health records, Information standards, Consolidated framework for implementation research, Consultation

## Abstract

**Background:**

Alcohol misuse is an important cause of premature disability and death. While clinicians are recommended to ask patients about alcohol use and provide brief interventions and specialist referral, this is poorly implemented in routine practice. We undertook a national consultation to ascertain the appropriateness of proposed standards for recording information about alcohol use in electronic health records (EHRs) in the UK and to identify potential barriers and facilitators to their implementation in practice.

**Methods:**

A wide range of stakeholders in the UK were consulted about the appropriateness of proposed information standards for recording alcohol use in EHRs via a multi-disciplinary stakeholder workshop and online survey. Responses to the survey were thematically analysed using the Consolidated Framework for Implementation Research.

**Results:**

Thirty-one stakeholders participated in the workshop and 100 in the online survey. This included patients and carers, healthcare professionals, researchers, public health specialists, informaticians, and clinical information system suppliers. There was broad consensus that the Alcohol Use Disorders Identification Test (AUDIT) and AUDIT-Consumption (AUDIT-C) questionnaires were appropriate standards for recording alcohol use in EHRs but that the standards should also address interventions for alcohol misuse. Stakeholders reported a number of factors that might influence implementation of the standards, including having clear care pathways and an implementation guide, sharing information about alcohol use between health service providers, adequately resourcing the implementation process, integrating alcohol screening with existing clinical pathways, having good clinical information systems and IT infrastructure, providing financial incentives, having sufficient training for healthcare workers, and clinical leadership and engagement. Implementation of the standards would need to ensure patients are not stigmatised and that patient confidentiality is robustly maintained.

**Conclusions:**

A wide range of stakeholders agreed that use of AUDIT-C and AUDIT are appropriate standards for recording alcohol use in EHRs in addition to recording interventions for alcohol misuse. The findings of this consultation will be used to develop an appropriate information model and implementation guide. Further research is needed to pilot the standards in primary and secondary care.

**Electronic supplementary material:**

The online version of this article (10.1186/s12911-018-0612-z) contains supplementary material, which is available to authorized users.

## Background

Alcohol misuse remains a major cause of preventable disability and death and disproportionately affects those living in the most socioeconomically deprived areas [1]. In 2015 there were 8758 alcohol-related deaths in the UK and the rate of alcohol-related deaths has remained unchanged in recent years [2]. Health and care services have a role in identifying alcohol misuse among patients and providing brief interventions [3]. This has been shown to be effective at reducing alcohol misuse particularly in men seen in primary and secondary healthcare services [4, 5]. It may even reduce mortality among heavy alcohol users admitted to hospital [5]. However, recording information about alcohol use using validated measures in both primary and secondary care remains poor [6, 7].

The UK Academy of Medical Royal Colleges (AoMRC) standards for the clinical structure and content of patient records recommends recording information about alcohol use in the social context of the medical history [8]. However, it does not specify how this information should be recorded and there are currently no widely endorsed and validated standards pertaining to this. Consequently, researchers and clinicians at the University of Birmingham and Queen Elizabeth Hospital Birmingham, in partnership with the Royal College of Physicians Health Informatics Unit, embarked on a project to develop information standards for recording alcohol use in electronic health records (EHRs) in the UK. The objectives of the standards were as follows:

- Enable healthcare staff and clinical information systems to identify patients at risk of alcohol misuse, and provide preventative and therapeutic interventions.
- Is relevant to public health and healthcare organisations to inform commissioning and delivery of preventative services and clinical audit of health promotion practices.
- Enables epidemiological and clinical research on alcohol consumption among patients in primary and secondary care.
- Enables patient-relevant information to be shared across the health and care system to improve coordination and continuity of care.

To inform the proposed standards, an evidence review was conducted on alcohol screening in secondary care (unpublished but available upon request) [9]. This review included 97 articles, which evaluated a total of 38 screening tests. This identified that the Alcohol Use Disorders Identification Test (AUDIT) [10, 11] and AUDIT-Consumption questions (AUDIT-C, which consists of the first three questions of the full AUDIT questionnaire) [12] were the most widely validated screening tools for alcohol misuse, and had been evaluated in a total of 26 and 16 studies, respectively. They both demonstrated a high uptake (75%, 95% CI 64–85% based on a meta-analysis of 10 studies) in a range of clinical settings as well as high sensitivity (ranging from 72 to 100%) and specificity (71–100% for AUDIT and 72–77% for AUDIT-C) for alcohol misuse. We therefore proposed that all patients accessing healthcare have their alcohol use recorded in EHRs using AUDIT-C as standard and that those with a high score complete the full AUDIT questions at some point in their care. We consulted stakeholders nationally to ascertain the appropriateness of the proposed standards and to identify potential barriers and facilitators to their implementation in practice.

## Methods

This was a qualitative analysis of responses to a national consultation on proposed information standards for recording alcohol use in EHRs. A consultation workshop was held at the Royal College of Physicians in London on 25th July 2016 to obtain the views of a wide range of stakeholders on the appropriateness of the proposed standards. Stakeholders were identified by the Royal College of Physicians Health Informatics Unit and the project team. They were invited from a range of relevant professional bodies (including medical royal colleges) and organisations representing patients and carers, clinicians, informaticians, public health specialists, and clinical information system suppliers. Attendees were provided background information on the proposed information standards and presented with the AUDIT and AUDIT-C questionnaires. They were asked to comment on their appropriateness as potential information standards for recording alcohol use in electronic health records and were asked five key questions (although participants were given the opportunity to comment beyond the scope of those questions):

1. How appropriate are the information standards?
2. What are the barriers to implementing the standards?
3. What are the facilitators to implementing the standards?
4. How might the standards be used to improve patient care?
5. Are there any patient safety issues with recording information in this way?

An online survey was launched on 17th January and closed on 05th March 2017 and disseminated to a wider range of stakeholders with the same questions and a number of additional questions that arose during the stakeholder workshop. A link to the consultation was also hosted on the Royal College of Physicians website. A full list of questions included in the online survey is provided in Additional file 1. All responses were anonymous, although basic information was captured about each respondent’s profession. The survey was disseminated to stakeholders via a number of different communication channels including targeted emails, bulletins, newsletters, and social media.

Responses from the survey were extracted onto an Excel spreadsheet and thematically analysed by a single researcher (SH) using the Consolidated Framework for Implementation Research (CFIR) [13]. The raw data and the validity of the coding was then checked by a second investigator (LM). This was followed by a discussion to reach consensus on the coding scheme and the mapping of the codes to the CFIR. While there is a subjective element to coding and that some of the themes could also be placed elsewhere or overlap with other constructs, a consensus was reached on the most suitable coding scheme. The CFIR was used as a pragmatic framework for constructing a mind map to visually conceptualise and organise the emerging concepts and themes. The CFIR was specifically chosen because it brings together a number of important conceptual frameworks for implementation research and was recommended by an external collaborator with expertise in implementation science. The CFIR has five main domains, each having a number of underlying constructs: intervention characteristics, inner setting (context within the organisation through which the process of implementation will take place), outer setting (external context in which an organisation sits), characteristics of the individuals involved, and the process of implementation.

## Results

### Participants

Thirty-one stakeholders participated in the stakeholder consultation workshop and 100 participated in the online survey (Table 1). This included patients and carers, healthcare professionals, public health specialists, informaticians, researchers, and clinical information system suppliers. Their responses to the consultation are summarised below, thematically grouped by the CFIR domains and constructs (Table 2). Selected quotes and a mind map of the thematic analysis are also provided in Additional files 1 and 2.

**Table 1:** Number of participants in the online survey by profession

Profession	*N*
Physician	51
General practitioner	7
Surgeon	5
Academic	4
Patient	4
Public health specialist	4
Midwife	3
Allied health professional	2
Data specialist	2
Healthcare manager	2
Homeopath	2
Nurse	2
System supplier	2
Alcohol trainer and consultant	1
Clinical informatician	1
Dental consultant	1
Dual Diagnosis Care Manager/Trainer	1
Healthcare commissioner	1
Paediatrician	1
Pharmacist	1
PhD student	1
Psychiatrist	1
Social enterprise founder	1
Total	100

**Table 2:** Summary of consultation findings

Domain	Construct	Description
Intervention	Relative advantage	Evidence based and validated
		Standardised and consistent
		Facilitate screening and brief interventions
		Diagnostic, prognostic, and social information
		Prescribing – drug interactions with alcohol
		Early recognition of alcohol withdrawal
		Temporal trends in alcohol use
		Audit, needs assessment, and research
	Adaptability – core components	Brief and simple
		User-friendly EHR interface
		Standard template
		Visual depiction of alcohol units
		Instant access to results and interpretation
		Frequency of recording is context dependent
		Lower AUDIT-C thresholds in pregnancy
		Age criteria
		Patient confidentiality
	Adaptability – adaptable periphery	Care pathways and support services
		Link with mental health services
		Wide range of health settings and health professionals potentially involved
		Self-completion of alcohol screening
		Direct patient access to EHRs and personal health records
		Inclusion in summary care records
		Electronic prompts for clinicians
	Other considerations	Costs and resources
		Piloting
Inner setting	Implementation climate	Integration with routine processes
		Clinical judgement
		Administrative burden
		Implementation of EHRs
		Integration of clinical information systems across health services
		IT infrastructure and digital connectivity
		Data governance
		Automation of care pathways
		Alignment with clinical coding standards and information models
		Organisational support and clear policy
		Clinical leadership
		Perceived importance among clinicians
		Financial incentives
		Key performance indicators
	Readiness for implementation	Training healthcare staff
		Implementation guide
		Access to EHRs
	Culture	Professional and cultural attitudes towards alcohol use
		Perception of usual practice
		Normalise alcohol screening and brief interventions in practice
	Networks, communication, and structural factors	Communication of benefits and relevance to clinicians and patients
		Sensitive and non-judgemental communication
		Clear information on care pathways and best practice
		Integration of alcohol and mental health services
Outer setting	Patient needs and resources	Underreporting of alcohol use
		Stigma
		Poor understanding of alcohol units
		Confidentiality
		Consent for data sharing between healthcare providers
		Association with poor mental health
		Adverse implications for life insurance, driving, and employment
		Bias future clinical assessments
	External policies and incentives	Clinical guidelines
		Alcohol health campaigns
		Low risk drinking guidelines
		Financial incentives
		Key performance indicators
		Labelling of alcohol units lacking
	Cosmopolitanism and peer pressure	Communication and data sharing between health services
		Coordination and continuity of care
		Influence of peers in primary care

### Intervention

#### Relative advantage

A number of respondents highlighted the importance of the information standards being evidence based and demonstrating patient benefit. A number of advantages were reported of the proposed standards over the current, unstructured approach to recording information about alcohol use, including improving the accuracy of estimating patients’ alcohol use. AUDIT and AUDIT-C were viewed as objective measures that can standardise and improve the consistency in the way alcohol use is recorded by health services. Implementation of the proposed standards could normalise the process of taking an alcohol history and familiarise clinicians with the appropriate questions to ask about alcohol use. This could also improve communication between healthcare providers and patients about alcohol-related risk. It could provide clinicians, patients, researchers, and policy makers with a shared definition and understanding of alcohol misuse.

Participants reported that the information standards could be used to improve the identification of patients at risk of alcohol misuse and improve access to relevant interventions and services. They could facilitate the implementation of alcohol screening and brief interventions in health services, the provision of general health promotion about alcohol use, and help link alcohol support services with patients. They could also help identify patients who are at risk of alcohol withdrawal and aid early prescription of alcohol withdrawal medication. The standards could potentially also improve access to mental health services and social care, and could be useful for identifying and managing cases of domestic violence, since alcohol misuse is an important risk factor.

Participants also commented that the standards could provide important diagnostic and social information and be useful for drug prescribing by highlighting potential drug-alcohol interactions. It may also have prognostic value by helping to estimate the risk of having or developing alcohol-related conditions. The quantitative nature of the proposed information standards (AUDIT-C or AUDIT score) would also enable access to temporal trends, which could help clinicians monitor alcohol use in their patients over time.

Implementation of the standards could improve the quality of data on alcohol use in EHRs. This would benefit epidemiological research and surveillance, allowing the derivation of more complete information on alcohol-related harms that could be accessed by patients, clinicians, public health specialists, and policymakers. The data could also be used for clinical audit, service evaluation, and quality improvement of alcohol support services.

### Adaptability

#### Core components

Participants described a number of core features that the information standards should meet. This included the need for the standards to be brief and simple, with particular consideration to how alcohol units are communicated and understood. The wording of the screening questions should be simplified as far as possible, particularly when self-completion by patients is expected. Respondents highlighted the importance of the EHR interface being user-friendly, with the standards embedded in a standard template that includes a visual display of alcohol units as well as the date of last entry. Clinicians using the template should also have instant access to the results and their interpretation.

A number of comments were also made about the frequency of recording alcohol use. Several respondents emphasised that the frequency should be evidence-based and not so frequent that patients are aggravated and disproportionate opportunity costs are incurred, or so infrequent that the information is out-of-date. Some felt that information about alcohol use should be updated at fixed, regular intervals, while others felt that the frequency should be dynamic, based on clinical judgement (e.g. where risks of alcohol-related harm have been identified or major life events have been reported), on the basis of previous scores, with higher scores requiring more frequent recording than lower scores, and based on age, with younger patients requiring more frequent recording than older patients. Respondents also felt it would be beneficial for clinicians to ask about alcohol use when prescribing medications that interact with alcohol, and also when making mental health assessments.

The frequency of recording was also felt to be dependent on the health setting and context. For acute hospital admissions, a range of opinions were expressed from recording information about alcohol use in all acute episodes to only recording information for overtly alcohol-related admissions, or where patients are previously known to have problems with alcohol misuse. Similarly, some respondents felt that alcohol use should be asked at all outpatient visits while others felt that it would only need updating annually or based on clinical judgement. In primary care, respondents varied in how frequently they felt that information on alcohol use should be asked, ranging from every 1 to 10 years, and also at NHS health checks.

There was more consistency in views about recording information about alcohol use among women attending antenatal appointments, particularly during the first booking visit. Respondents felt thereafter that pregnant women could be asked about their alcohol use based on the clinical judgement of their midwife, at every antenatal visit, or specifically around the second or third trimester of pregnancy. Respondents also felt that AUDIT-C score thresholds should be lowered in pregnancy to align with the advice to be teetotal during pregnancy.

A number of comments also concerned the eligible population that the standards should apply to. Generally respondents felt that they would be appropriate for patients aged 16 years and older and potentially in those aged 10–16 years where alcohol misuse was being suspected, although this would require sensitive handling and would entail issues around obtaining parental consent. The standards were felt to be inappropriate for patients under the age of 10 years, during end-of-life care, and for patients with learning difficulties. Respondents also expressed the importance of ensuring patient confidentiality and where possible providing a private consultation setting when asking patients about their alcohol use.

#### Adaptable periphery

Some respondents emphasised the importance of having a whole systems approach to reducing alcohol-related harm, of which the information standards would form one component. The information standards were generally only considered to be useful in the presence of appropriate care pathways and support services. This included the availability of alcohol liaison specialists and support workers, proactive signposting of patients to support services, provision of appropriate patient information, a clear process for management and referral, and for mental health assessment and treatment. Some respondents therefore felt that the information standards should incorporate some items related to recording the management of alcohol misuse.

A number of health settings were considered appropriate for the implementation of the standards including general practice and outpatient departments, with particular emphasis on making use of waiting rooms to gather relevant information prior to appointments. Other suggested settings included emergency care, medical and surgical assessment units, inpatient hospital wards, antenatal clinics, mental health services, pharmacies, sexual health clinics, dental practices, community nursing, addiction services, social care, health visiting, school nursing, paramedics, and paediatric services. Respondents generally felt that nurses, junior doctors, GPs, alcohol support workers, consultants, healthcare assistants, midwives, and even non-health professionals (e.g. social workers) had a responsibility for collecting information about their patient or client’s alcohol use.

A number of respondents expressed the importance of patients being able to directly provide information about alcohol use, either by having access to their EHRs or personal health records, [14] or by paper-based questionnaires (including postal) or scratch cards that would later be entered onto their health records. Patient access to EHRs would require the development of appropriate online digital platforms (e.g. web portals or smartphone apps), and could include use of tablet computers or fixed computer terminals in waiting rooms. Digital platforms could be designed to help patients estimate their alcohol units and completion of the standards by patients could be encouraged through digital prompts. However this would require a degree of digital skills which not all patients would necessarily have and all modalities would require strict data protection and protect patient confidentiality.

Most respondents to the online survey agreed that the information standards should be incorporated into summary care records, provided that patient confidentiality was sufficiently protected. The summary care record is a copy of key information from the primary care record. It provides authorised care professionals with faster, secure access to essential information about patients when they need care. A smaller majority of respondents felt that the standards could be included in discharge summaries, although this was felt to be dependent on gaining patient consent, and when alcohol misuse was deemed to be relevant to the presenting problem, and was specifically identified during the clinical episode. Some respondents also felt that clinical information systems could be programmed to prompt healthcare providers to ask patients about alcohol use.

#### Other considerations concerning the intervention

Participants also commented on considering the costs of implementing the information standards, including the time taken for staff to ask about alcohol use, the resources needed to train staff on alcohol screening and brief interventions, and the cost of employing staff to provide alcohol support and associated administrative support. Respondents also highlighted the costs that would be associated with updating and adapting multiple clinical information systems with the information standards. Some also suggested that the standards could be piloted to ensure their feasibility in practice.

### Inner setting

#### Implementation climate

A number of issues were raised about the implementation of the information standards in health settings. This principally concerned integration with routine processes, IT systems and infrastructure, and administrative burden. Respondents felt that the standards were more likely to be successfully implemented if completion was integrated with the admission pathway in secondary care, including nursing and medical clerking. In primary care, it was generally felt to be appropriate to include completion of the standards as part of GP registration. There was also felt to be an important role for clinical judgement in determining when it would be appropriate to ask about alcohol use and complete the screening questions. The additional administrative burden associated with capturing information about alcohol use and making referrals to alcohol support services would also have to be accounted for when planning the implementation of the standards.

IT systems and infrastructure were seen to be key factors that would determine the likely success of implementing the alcohol information standards in practice. This included the degree of implementation of EHRs, the integration of clinical information systems and shared EHRs across all elements of the health system (avoiding duplication of data entry), access to EHRs using mobile devices such as tablet computers, and ready patient access to EHRs and personal health records [14] to enable self-completion of information about alcohol use. Respondents also highlighted the importance of digital connectivity in health settings, as well as data security and governance (requiring patient consent for data sharing), standardisation of clinical coding, and the development of clinical information systems to automate referrals and signposting to support services. Variability in the current coding of alcohol use was seen as a barrier to understanding patient need. Some respondents also added that it was important to ensure the information standards were compatible with existing clinical coding standards and information models such as OpenEHR [15] and the national maternity and cancer information sets [16, 17].

A number of cultural and organisational factors that would influence implementation were also highlighted. This included the relative priority given by an organisation for delivering alcohol screening and brief interventions. Implementation of the standards would require organisational support, clinical leadership, and the provision of a clear policy for asking patients about their alcohol use using the recommended standards, as well as providing appropriate interventions. This would also depend on the perceived importance among clinicians of alcohol misuse in their patients and their role in preventing alcohol-related harm. Clinical champions potentially have an important role in fostering the clinical engagement needed for successful implementation in practice.

A number of organisational incentives and rewards were identified that might influence the implementation of the standards. Two key financial incentives include the Quality and Outcomes Framework (QOF) in primary care, [18] and Commissioning for Quality and Innovation (CQUIN) in secondary care [19]. Both are financial incentive schemes for achieving specific healthcare processes and outcomes in England. There are currently no QOF outcomes specifically related to recording alcohol use (except in the context of bipolar and psychotic disorders) or delivering brief interventions. However, the most recent CQUIN for England includes outcomes specific to alcohol screening and providing brief advice or referral in hospitals [19]. Some respondents suggested that implementation of the standards could also potentially be facilitated by healthcare organisations making them a mandatory requirement or by including related outcomes as part of their key performance indicators.

#### Readiness for implementation

A number of factors were reported by participants that could influence the readiness for implementation of the information standards in healthcare organisations. Respondents highlighted the importance of access to relevant information and knowledge, including healthcare staff having access to training on alcohol identification and brief advice. In order to implement the standards in practice, healthcare staff will need to have the necessary knowledge, skills, and confidence to ask patients about their alcohol use. An implementation guide was also viewed as important to provide operational clarity and should be targeted and adapted for both clinical information system suppliers and healthcare providers. Respondents also commented on the importance of healthcare staff having the necessary level of digital literacy to use EHRs effectively in their practice and to have ready access to EHRs so that information about alcohol use can be recorded with relative ease.

#### Culture

Respondents felt that professional and cultural attitudes towards alcohol could influence the successful implementation of the data standards. Alcohol consumption and misuse among health professionals could plausibly reduce the likelihood of them asking their patients’ about alcohol use. Similarly clinicians’ perception of what constitutes usual practice, and viewing alcohol screening as beyond that, could act as a barrier to implementing the standards. Conversely, successful implementation of the standards in routine practice could help normalise alcohol screening and brief interventions in health and care services.

#### Networks, communication, and structural factors

Participants stressed the importance of clearly communicating the benefits of alcohol screening and brief interventions to both clinicians and patients. Patients would need to be explained why they were asked about alcohol use when presenting with unrelated (or seemingly unrelated) conditions, and that the information should be asked in a sensitive and non-judgemental way. Clinicians would also need to be informed of the available care pathways and guidance on best practice. A number of participants also expressed that implementation of the standards could be facilitated by better integration of alcohol and mental health services.

### Outer setting

#### Patient needs and resources

Participants raised several patient-related factors that might influence the implementation of the information standards. A number highlighted the stigma associated with alcohol misuse and the importance of patient confidentiality. Patients may underreport their alcohol use for this reason but also because of a poor understanding of alcohol units. Conversely, implementation of the standards could help patients gain a better understanding of their level of alcohol use in relation to national guidelines [20]. The way information about alcohol use is ascertained will need to be acceptable to patients and culturally sensitive. Consent would need to be obtained for sharing this information with other healthcare providers. Participants also reported that healthcare providers would need to consider mental health when assessing patients for alcohol misuse since alcohol misuse frequently occurs in the context of poor mental health.

A number of potential adverse impacts were also identified from implementing the standards. Participants reported that information about alcohol misuse could have implications for life insurance, medical reports for the Driving and Vehicle Licence Authority (DVLA), prospects for employment, and interactions with the criminal justice system. If patients objected to having this information asked and recorded in their health records, this might negatively impact on their relationship with clinical services. Records of alcohol misuse could also potentially bias future clinical assessments towards diagnosing alcohol-related disorders.

#### External policies and incentives

A number of external policies and incentives that could influence the implementation of the alcohol information standards were highlighted. The National Institute for Health and Care Excellence (NICE) guidelines on prevention of alcohol-use disorders, [3] the NHS Digital Strategy, [21] and alcohol-related health campaigns, were all seen as potential facilitators. Similarly, implementation of the standards could help put the NICE guidelines on prevention of alcohol-use disorders into practice. Participants also felt that the standards should align with the UK Chief Medical Officers’ low risk drinking guidelines [20]. Incorporation of outcomes on alcohol screening and brief interventions in key performance indicators for healthcare organisations could promote use of the standards, as could financial incentives such as the national CQUIN in England [19], as well as developing similar financial incentives for primary care. Beyond the context of health and care services, the lack of clear labelling of alcohol units on alcoholic beverages was seen as a barrier to implementation of the standards, since this impeded public understanding of alcohol intake and recommended low risk limits.

#### Cosmopolitanism and peer pressure

Implementation of the information standards could also be influenced by, or potentially facilitate communication and data sharing about alcohol use between primary care, secondary care, public health and other elements of the health service. This could potentially improve coordination and continuity of care for patients with alcohol misuse and facilitate an integrated approach to preventing further harms. Finally, awareness of local general practices actively implementing the standards and providing alcohol screening and brief interventions in primary care could potentially influence GPs to implement the standards in their own practices.

## Discussion

### Main findings

In this national consultation of 131 stakeholders, participants broadly agreed that recording the AUDIT-C and the full AUDIT questionnaire for patients with a high AUDIT-C score, is an appropriate standard for recording information about alcohol use in EHRs. Participants also agreed that the standard should include some items about the management of patients identified as at-risk of alcohol misuse. Implementing this standard could improve the early identification of alcohol misuse, the delivery of brief interventions, and access to specialist services. They could also have a number of secondary uses for improving service quality, healthcare commissioning, and epidemiological research.

A number of factors were identified that are likely to influence the implementation of the standards in practice. In particular, the standards will need to be brief and simple and embedded within EHRs as a standard template with a visual illustration of alcohol units and will require clear care pathways for individuals identified with a high risk of alcohol misuse. The frequency of recording information about alcohol use will be context dependent and will vary by setting and prior risk. The standards are likely to be applicable in a wide range of health settings for patients aged 16 years and older and potentially in those aged 10 to 16 years where alcohol misuse is suspected.

The standards would work best if they were communicated effectively between primary and secondary care (through summary care records) as well as other relevant aspects of the health and care service to provide a more integrated approach to managing and preventing alcohol misuse. However, information sharing across the health service will require robust data governance and acquisition of patient consent. Implementation of the standards will require additional healthcare resources to cover training, support services, and development of clinical information systems.

Implementation of the standards could be facilitated by integrating them with existing clinical pathways, improving the accessibility of EHRs, and by providing training on alcohol screening and brief interventions, as well as a clear implementation guide. Organisational support and clinical engagement, implementation of relevant NICE guidelines, [3] and external financial incentives, would also encourage the implementation of the standards. The implementation strategy will also need to consider the stigma associated with alcohol misuse, the importance of ensuring patient confidentiality, under-reporting of alcohol use, and the cultural attitudes towards alcohol among both patients and health and care professionals.

### Relationship to other studies

An analysis of free-text documentation of alcohol use in the social context module of EHRs in an academic hospital in the USA found that users had difficulty documenting alcohol use frequency, amount, and status within structured fields [22]. Free-text descriptions of frequency were commonly used, using variable terms and a significant proportion of those with suggestions of alcohol misuse did not have this documented in their past medical history or problem list. The authors highlighted the importance of improving clinical information systems, user training, decision support, and information standards to improve the documentation of alcohol use in EHRs. Participants in our consultation similarly reported that recording information about alcohol use in clinical records was currently highly variable and unstructured and that the development of standards could help improve the consistency of recording. Furthermore the quantitative nature of AUDIT-C and AUDIT should help clinicians record information about quantity and frequency of alcohol consumption using a more structured and validated approach.

A systematic review and meta-analysis of the clinical recognition and recording of alcohol disorders in primary care found that GPs were able to identify alcohol use disorders in 42% of cases, but recorded this correctly in only 27% of primary care records [23]. Secondary care clinicians were found to identify alcohol use disorders in 52% of cases and recorded this correctly in only 37% of medical records. The authors recommended considering the use of simple screening methods rather than relying solely on clinical judgement for identifying alcohol-related problems. This aligns with views expressed by stakeholders in our consultation that a more systematic approach is needed to identify alcohol misuse and fits with the proposed recommendations to use AUDIT-C and AUDIT as screening questions to incorporate within EHRs as the standard approach to asking patients about alcohol use.

### Strengths and limitations

A large number of stakeholders from a wide range of relevant professions (including patients and carers) and organisations were consulted to inform the proposed standards for recording information about alcohol use in EHRs. The Consolidated Framework for Implementation Research was used to thematically analyse the responses, which provided a predefined and validated conceptual framework for organising the findings.

However, the findings of this consultation are prone to response bias since the stakeholders who accepted our invitation to participate are likely to have an interest in preventing and managing alcohol misuse and related disorders. The views expressed by the participants may therefore not necessarily reflect those of all patients, clinicians, and service providers. Furthermore, the majority of participants were physicians, which is likely due to the consultation being hosted by the Royal College of Physicians. The interpretation and analysis of the responses may also have been influenced by the authors who are all involved in a national project to develop information standards for recording alcohol use in EHRs. Finally, the proposed information standards were not evaluated by stakeholders against all the implementation considerations that arose from this consultation. This would be worth considering for future research. In addition, future work could assess the views of stakeholders on the relative utility of alternative screening tools for alcohol misuse.

### Implications for policy, practice, and research

The findings of this consultation support use of AUDIT-C and AUDIT as information standards for recording alcohol use in EHRs. The findings will be used to develop an appropriate information model that can be used by system suppliers to implement the standards in clinical information systems and to develop an implementation guide for clinicians, service providers, and system suppliers. The proposed standards will need to be piloted in primary and secondary care to ensure that it can be feasibly implemented in real world practice and linked to appropriate care pathways. Further research will also be needed to evaluate how they can be best used to systematise the delivery of brief interventions and specialist referral for alcohol support in health and care services.

## Conclusions

A wide range of stakeholders agree that use of AUDIT-C is a useful standard for recording information about alcohol use in EHRs among patients accessing health services and that AUDIT should be used for further risk assessment in those identified as at-risk of alcohol misuse. Further work is needed to incorporate the findings of this consultation into an information model and implementation guide and to pilot the proposed standards in primary and secondary healthcare settings.

## Additional files

Additional file 1: This includes the survey questions and selected quotes (DOCX 33 kb)

Additional file 2: This is a mind map of the key themes identified in our consultation mapped against the domains of the Consolidated Framework for Implementation Research (CFIR). (PNG 1468 kb)

## Abbreviations

AoMRC: Academy of Medical Royal Colleges
AUDIT: Alcohol Use Disorders Identification Test
AUDIT-C: Alcohol Use Disorders Identification Test-Consumption
CFIR: Consolidated Framework for Implementation Research
CQUIN: Commissioning for Quality and Innovation
DVLA: Driving and Vehicle Licence Authority
EHR: electronic health record
IT: Information technology
NICE: National Institute for Health and Care Excellence
QOF: Quality and Outcomes Framework
