# Supplementary material for: Information standards for recording alcohol use in electronic health records: findings from a national consultation
Source: BMC Med Inform Decis Mak. 2018 Jun 7;18:36. doi: 10.1186/s12911-018-0612-z (PMC5992754; doi:10.1186/s12911-018-0612-z)
Supplement: Supplementary file 1 — This includes the survey questions and selected quotes (DOCX 33 kb) [file 12911_2018_612_MOESM1_ESM.docx]

Supplementary file

# Survey questions

1. How appropriate is the proposed information standard with respect to the stated objectives?
2. What are the barriers to implementing the information standard in electronic patient records?
3. What would help facilitate the implementation of the information standard in electronic health records?
4. Are there any patient safety concerns with collecting information about alcohol consumption in this way?
5. How can using a standard way of recording alcohol consumption in electronic health records be used to improve patient care?
6. How frequently should information on alcohol consumption be updated in an electronic health record?
7. Which healthcare professionals would be well placed to record information on alcohol consumption in electronic health records?
8. How can patients be enabled to provide information on their alcohol consumption to update their electronic health record?
9. What would be sensible and feasible clinical settings to ask patients about their alcohol consumption?
10. What age range of patients should the information standards be applied to?
11. Should the information standard be modified in any specific clinical contexts or for specific patient groups?
12. Should the information standard be incorporated in the Summary Care Record?
13. Should the information standard be incorporated in discharge summaries?

# Selected quotes

## How appropriate is the proposed information standard with respect to the stated objectives?

*“This is potentially very useful as we know that current documentation of a quantitative alcohol history is very poor in medical notes... I would suggest it is essential to ensure there are pathways in place to process the patients identified as being "at-risk", so that existing alcohol treatment services are not swamped.”* (Physician)

*“Although this test could provide good quality information, I am concerned that is yet another additional piece of information to include in the patient assessment. It is unlikely to be properly done.”* (Physician)

*“A screening test should be one or two sensitive questions max. Cage is better because it is simpler. This is too complex and will result in clinical disengagement and poor data quality. Better to keep the screening test as simple as possible.”* (Physician)

*“Very appropriate. The initial AUDIT-C^[[1]](#footnote-1)^ questions can be asked by any healthcare professional; the follow up questions are more time consuming and in my Trust are asked by the Drug and Alcohol Liaison nurse.”* (Physician)

*“Enabling patient-relevant information to be shared across the health system, in this context, is often viewed as a breach of confidentiality by the patient. We often inform GPs that people are in treatment and their level of alcohol consumption as it forms part of their treatment agreement, but anything beyond that may be inappropriate.”* (Public health specialist)

*“This data set is absolutely appropriate and will form a solid foundation to understanding the needs of an individual as well as population wide, to help commission preventative and therapeutic interventions.”* (Public health specialist)

*“It seems appropriate but may be impractical - at least at present where it is uncommon for patients to be given the means of inputting data into their medical record.”* (Surgeon)

## What are the barriers to implementing the information standards in electronic health records?

*“IT issues, time constraints, lack of training and engagement of staff…”* (Midwife)

*“Administrative staff and GP's who may misunderstand its purpose and feel it is an additional and superfluous administrative requirement and therefore resource cost. Potential difficulty with IT/system rollout; patients who may consider it intrusive and be concerned about data sharing…”* (Patient)

*“…time, confidence to ask questions, professionals’ own use of alcohol and the value judgement they may hold about alcohol…”* (Public health specialist)

*“No services or inadequate community services…”* (Physician)

*“Probably easy enough in some settings, but not in others. In secondary care I would imagine that whether the tool was used would depend on staff recognising the value of using it.”* (Patient)

*“Patient under-reporting…”* (Patient)

*“…low priority and poor awareness of the scale of alcohol related harm…”* (Physician)

*“Wide variation in hospital IT systems. Perceived time pressures in the acute corridor (although alcohol screening can generally be accomplished very quickly). Resistance to change amongst staff.”* (Physician)

*“We do not have electronic records.”* (Physician)

*“There is a multitude of tick box assessments in our electronic system-any additional ones need to be short and allow a minimum data input, or data will be inaccurate.”* (Physician)

*“The burden of completing mandatory electronic screening tools, and increasingly now the investigation of the incident reports generated if they are omitted, is stressing nursing staff.”* (Physician)

*“Access to secure electronic systems.”* (Physician)

*“Community health services may not always have access to patient record and would have to record AUDIT-C and transfer to patient records through another service such as primary care.”* (Public health specialist)

*“Pressure on time in teams makes complex data collection like this challenging. This may also discourage some service users from engaging with us, and could possibly lead to discrimination.”* (Allied health professional)

*“…if the patient has a significant mental illness and thus may use alcohol to try to manage said illness our local mental health team won't accept referral because they are alcohol dependent!”* (GP)

*“Mental health always say the client must be sober before they can begin any work with them -but often they are using alcohol to 'self-medicate' their mental health issues”* (Public health specialist)

*“…understanding of alcohol units…”* (Academic)

*“…lack of mobile electronic devices is a barrier.”* (Midwife)

*“In all health settings, having appropriate trigger points so that data is collected at the right place in the clinical workflow”* (Pharmacist)

*“Stigma”* (Not-for-profit organisation)

*“General lack of organisational support and buy-in to standards including training, resources and reflection on practice. Limited national leadership.”* (Alcohol trainer/consultant)

*“There has to be a clear pathway for any professional obtaining a score to refer onwards, otherwise it is pointless.”* (Paediatrician)

*“The key barrier would be IT/electronic related. Patient's digital literacy may have to be considered so additional support may be required for patients.”* (Patient)

*“The ability to have access to shared records across systems.”* (Physician)

*“…healthcare settings expecting payment to do AUDIT^[[2]](#footnote-2)^ and it not being seen as part of the day job.”* (Public health specialist)

## What would help facilitate the implementation of the data standard in electronic patient records?

*“Provision of standardised software.”* (Academic)

*“Clear explanation about the aims and objectives of use of data and no adverse effect to patient in providing the information.”* (Allied health professional)

*“Data input templates, ability to print off questionnaire and persuading GPs there was a real benefit in using it.”* (GP)

*“Money. Attach it to a QOF^[[3]](#footnote-3)^ standard and it may work…”* (Healthcare commissioner)

*“NICE Guidance. Evidence on value of information and relevance to care.”* (Healthcare manager)

*“Access to mobile electronic devices.”* (Midwife)

*“Clear policy as to who should be asked and how often.”* (Clinical informatician)

*“Knowledge that other practices were using it and the availability of alcohol liaison specialists for referral.”* (Patient)

*“Incorporate into GP registration questionnaire.”* (Physician)

*“Education and awareness.”* (Physician)

*“Training, systems that make data entry easier, instant access to interpretation of results, time to do this.”* (Public health specialist)

*“CQUINs^[[4]](#footnote-4)^ appear to have proven effective in some areas…”* (Alcohol trainer/consultant)

*“Evidence that use of the standard had an impact on outcomes.”* (Patient)

*“Make it a mandatory part of admission or hospital stay.”* (Patient)

*“The alcohol screening would need to be compulsory - our experience and others have shown that where screening remains optional, it is often missed by the staff. Making screening compulsory for electronic admission would greatly help.”* (Physician)

*“Good user interface on EPR.”^[[5]](#footnote-5)^* (Physician)

*“Patient to do it themselves in waiting room rather than in front of HCP.”* (Physician)

*“Incorporate into clinical systems from different suppliers. Keep it as quick and simple as possible to use.”* (Physician)

*“Pull data from GP record / SCR,^[[6]](#footnote-6)^ and allow clinician to amend/correct it rather than entering it de novo.”* (Physician)

*“Being very clear about what the next step is and how exactly it helps. Similar to dementia screening - it doesn’t get done because no one feels confident it actually makes the patient’s life better.”* (Physician)

*“For some patients, self-completion could be tried (linked to feedback of score and explanation/advice).”* (Physician)

*“Self-completion methods for example using tablets to enable clients to answer these screening questions whilst waiting prior to the appointment to enable clinicians to do second questionnaire (when relevant) in the appointment.”* (Allied health professional)

*“Training on alcohol units for those who will help patients fill in the AUDIT-C.”* (Academic)

*“Demonstrate that it is practical and valuable in each care setting, and that it would not be repeatedly requested of the patient once it has been filled out once.”* (Data specialist)

*“Get secondary care and community care records on computers which can link with primary care.”* (GP)

*“An app for patients to complete the questions at their leisure.”* (GP)

*“In all settings, provision of implementation guidance to suppliers, so they understand how the data standard works and what it is trying to achieve.”* (Pharmacist)

*“Healthcare professionals offering to assist patients with completion will lead to more accurate completion and patient accuracy of reporting.”* (Patient)

*“The recording of data from the patient through a portal that will feed into the organisation's EPR and therefore provide the relevant results.”* (Patient)

## Are there any patient safety concerns with collecting information about alcohol consumption in this way?

*“…what do you do with the answers when there are few services to offer? Has adverse impact on medical reports requested by DVLA,^[[7]](#footnote-7)^ insurance companies and work/ occupational health.”* (GP)

*“The data will inevitably be shared and may be out of date and is fundamentally inaccurate due to under reporting, leading to the patient being misjudged and potentially mistreated.”* (GP)

*“Only that if done as a data collection exercise it will increase patient distancing from the service and make engagement with alcohol reduction harder so the point of the data collection must be that it is part of improving clinical care.”* (GP)

*“…safeguards against identification need to be put in place if there are risks to anonymity…”* (PhD student)

*“Many primary healthcare practices appear to be collecting AUDIT information but offering no feedback/intervention. As well as a waste of money, this is a neglect of duty of care - people may assume no feedback means 'all good'.”* (Alcohol trainer/consultant)

*“Not everyone is honest about the amount they consume or how often. Some patients will not even realise they are consuming large amounts on a regular or binge basis.”* (Patient)

*“Consideration and advice should be given around the safety of data sharing and patient privacy. Patients with a history of excess drinking may be extremely concerned about any implications being made public, whether in relation to their work, their relationships or their family or community life. Health settings need to take electronic security and online privacy and confidentiality extremely seriously as to not do so may actually undermine patient trust in health settings and cause a patient to stop seeking help.”* (Patient)

*“If doctors are asked to do this they will have less time.”* (Physician)

*“Police or insurance companies may be interested in confidential data.”* (Physician)

*“Diversion of resources away from the physicians’ main aim of diagnosis and treatment.”* (Physician)

*“The label of "alcohol misuse" is difficult to shake off, even after years of abstinence, and can lead to diagnostic biases.”* (Physician)

*“This may not be acted on, the services to help to reduce alcohol consumption may not exist and the evidence to support alcohol reduction may not be strong…”* (Surgeon)

## How can using a standard way of recording alcohol consumption in electronic patient records be used to improve patient care?

*“It would be an excellent resource for epidemiological research, which might then be used to keep patients better informed about the health risks associated with drinking.”* (Academic)

*“It may of course act as a reasonable prompt for clinicians to act.”* (Academic)

*“Information may affect decisions on the management of the patient and any follow up support they may require.”* (Allied health professional)

“Improve treatment in mental health by more appropriate targeting.” (Allied health professional)

*“When this is done (and it is aimed for in GP!) it is then easy to see trends; research is also easier.”* (GP)

*“The correct services would be provided. Further, needs such as depression and other mental health issues could well be identified as underlying causes.”* (Healthcare commissioner)

*“Electronic patient records are supposed to be visible to all who need access so the whole of a patient's needs are met. This means all health professionals should be able to see information pertaining to each patient's care. It may help to predict more accurately those who may develop alcohol related health conditions. It may help heavy drinkers to realise the harm they are doing to themselves and others. It may help with the prescribing of medications.”* (Healthcare manager)

*“More patients at risk of harm from alcohol could be identified and then offered advice and support.”* (Midwife)

*“Audit, service evaluation, raise awareness and give patients permission to discuss any alcohol issues.”* (Midwife)

*“This is already a standard question used within the maternity service; however the way in which information is collected may vary - having a standardised format would ensure that there is more consistency and women with alcohol problems are more likely to be identified and the appropriate interventions put into place.”* (Midwife)

*“This standard can be used by clinicians to prompt patients to reflect on their drinking behaviour, and consider what changes they are willing to make.”* (Pharmacist)

*“Policy makers, clinicians and researchers will be speaking about the same thing when they refer to a term indicating alcohol use or misuse.”* (PhD student)

*“By making it easier to identify people who are at risk and then monitoring them.”* (Clinical informatician)

*“It can be the trigger for a brief intervention but some training is needed to ensure that the brief intervention happens (and is the right intervention).”* (Paediatrician)

*“Providing a record that is accessible to all healthcare professionals should make it easier for them to be aware when organ damage occurs and to act appropriately. It is necessary to have support for patients who need it to modify their drinking habits.”* (Patient)

*“Raises awareness by health care professionals of patients' problematic alcohol use. Can be crucial with respect to diagnosis and treatment e.g. cardiac arrhythmia.”* (Patient)

*“Through simplification it should demand less of the patient and those who support as well as those who record, administrate and make clinical decisions based on the information generated. It should stimulate patients to think more specifically about their own individual drinking patterns and so mainstream behaviour.”* (Patient)

*“Patients drinking to potentially dependent levels will be identified and offered a referral to alcohol liaison nurse services.”* (Physician)

*“In secondary care, this can identify patients at highest risk of alcohol withdrawal and medical consequences. It also identifies patients whose alcohol misuse may not be routinely detected (e.g. if admitted with a condition not thought typically related to alcohol).”* (Physician)

*“Once completed, it could automatically highlight the fact that someone is at high risk and suggest referral.”* (Physician)

*“It will give a more accurate estimate of the size of the problem and the response required.”* (Physician)

*“Identify at-risk individuals and offer help/intervention before disease develops.”* (Physician)

*“It will flag up those at high risk and may influence medical treatments.”* (Physician)

*“If scoring >5 a flag to notify clinician comes up, then advice might be given.”* (Physician)

*“Record accurate population data to guide public health. Share information across professionals involved in care of individuals.”* (Physician)

*“Sharing standard information improves care, especially emergency care when the patient cannot always express their alcohol consumption.”* (Physician)

*“Assuming it is accurate and reproducible, it helps identify at risk behaviour in whom behaviour modifying interventions can be targeted.”* (Physician)

*“Better understanding of alcohol consumption for patients and carers will help target prevention strategies where they are needed most.”* (Physician)

*“Would give patients an objective measure of their alcohol consumption.”* (Physician)

*“Could be used as an audit tool for identifying risks for disease management, give information to support continuing commissioning of alcohol support services.”* (Physician)

*“Flags up any concerns regarding alcohol consumption and drug interactions…”* (Physician)

*“If used appropriately in acute situations, it should reduce risk of unmanaged alcohol withdrawal and its associated severe morbidity. In non-acute settings such as inpatient rehabilitation, staff often build trust with patients and could use the information to promote lower-risk lifestyle.”* (Physician)

*“Trigger an appropriate response in terms of alcohol withdrawal meds, health information, something in the discharge summary for GP and patient.”* (Physician)

*“Appropriate trigger for referral to alcohol addiction services/alcohol liaison nurses.”* (Physician)

*“Accurate information regarding alcohol consumption minimises risk with regard to prescribing. Accurate information regarding alcohol consumption ensures the interventions provided are appropriate…”* (Public health specialist)

*“The standardised data set would provide a consistent picture across the population, enabling consistency in the response and services provided to the population. Consistency is also likely to raise more awareness amongst the public as it will become a familiar and common process.”* (Public health specialist)

*“To help identify where further structured support is needed, to identify alcohol issues earlier when more can be done to address harms or prevent further harms.”* (Public health specialist)

## How can patients be enabled to provide information on their alcohol consumption to update their electronic health record?

*“Use new IT systems with interactive app to record consumption.”* (Allied health professional)

*“Modify patient access to personal health record. Have the pro-forma linked to this site and allow patients to self-complete. Perhaps if score suggests high risk then suitable advice sheets and info on where to get help should automatically pop up.”* (GP)

*“This would have to be through a medical professional at the moment, and to each acute and community service they require as there is a lack of sharing of data. Ideally they should be able to log in to an IT system and complete a short form to update, or a form from the GP.”* (Healthcare commissioner)

*“Give information about risks of alcohol and a form to complete prior to being seen.”* (Midwife)

*“Patient education, public health advertising like Balance North East. Audit C is a good tool because it gives examples of units of alcohol. Private consultation room for confidentiality. Active listening skills and training for staff.”* (Midwife)

*“Need to have access in order to be able to do this. In maternity we work in partnership with women and they take ownership of the hand held record for the duration of the pregnancy - this needs to be extended out with the move to digitalising this.”* (Midwife)

*“Make it routine and non-threatening.”* (Psychiatrist)

*“Questioner being open, honest, tactful, sympathetic and non-judgemental.”* (Homeopath)

*“Build a smartphone app that lets people score themselves and can email the score to the relevant person (GP, hospital secretary). Or have direct entry using a tablet or similar at the clinical encounter.”* (Paediatrician)

*“Communication must be adult to adult and without judgement. It must be made plain to them that this information is held in strict confidence.”* (Patient)

*“A positive spin needs to be put on the collection of information and the more communication with the patient the more likely they are to give accurate information.”* (Patient)

*“Digital skills training particularly on tablets and smartphones, via email reminders and hyperlinked texts, be reminders being sent to appropriate family members, case workers and those who have a regular trusted relationship with the patients concerned. To actively use tablets and fixed terminals in outpatients surgeries, general practice and in other community settings such as dental surgeries, libraries, walk in health centres, and council offices.”* (Patient)

*“AUDIT-C scratch cards have been used successively in our admissions unit.”* (Physician)

*“Given limited understanding of alcohol units (by healthcare staff as well as the general public) this needs to be standardised e.g. by use of visual cues for the healthcare staff or patient.”* (Physician)

*“Providing iPads or similar gadgets in the waiting room.”* (Physician)

*“Leaflets describing alcohol units would be helpful.”* (Physician)

*“When joining a General Practice. By completing a questionnaire, either on paper or tablet when attending for a consultation.”* (Physician)

*“Access to patient areas of health records online or via an app.”* (Physician)

*“Online through access to their GP records.”* (Physician)

*“Provide computer access to smoking/drinking questionnaires when booking in for routine GP/hospital appointments.”* (Physician)

*“They could update a Personal Health Record but they don't really exist at scale.”* (Physician)

*“Given a physical copy of the AUDIT / AUDIT-C to take away and return. The accuracy of the reporting is likely to have increased because they can answer the questions in a "non-judgemental" environment where they feel comfortable.”* (Public health specialist)

*“They could interact with electronic record access where they can update and keep drink diaries which could be linked back to their EPR.”* (Public health specialist)

*“Integrate with patient wellness apps. Allow patients access to their patient records and update it from home/work.”* (System supplier)

1. Alcohol Use Disorders Identification Test-Consumption [↑](#footnote-ref-1)
2. Alcohol Use Disorders Identification Test [↑](#footnote-ref-2)
3. Quality and Outcomes Framework [↑](#footnote-ref-3)
4. Commissioning for Quality and Innovation [↑](#footnote-ref-4)
5. Electronic Patient Record [↑](#footnote-ref-5)
6. Summary Care Record [↑](#footnote-ref-6)
7. Driving and Vehicle Licensing Authority [↑](#footnote-ref-7)
