# Supplementary figures and images for: Information standards for recording alcohol use in electronic health records: findings from a national consultation
Source: BMC Med Inform Decis Mak. 2018 Jun 7;18:36. doi: 10.1186/s12911-018-0612-z (PMC5992754; doi:10.1186/s12911-018-0612-z)

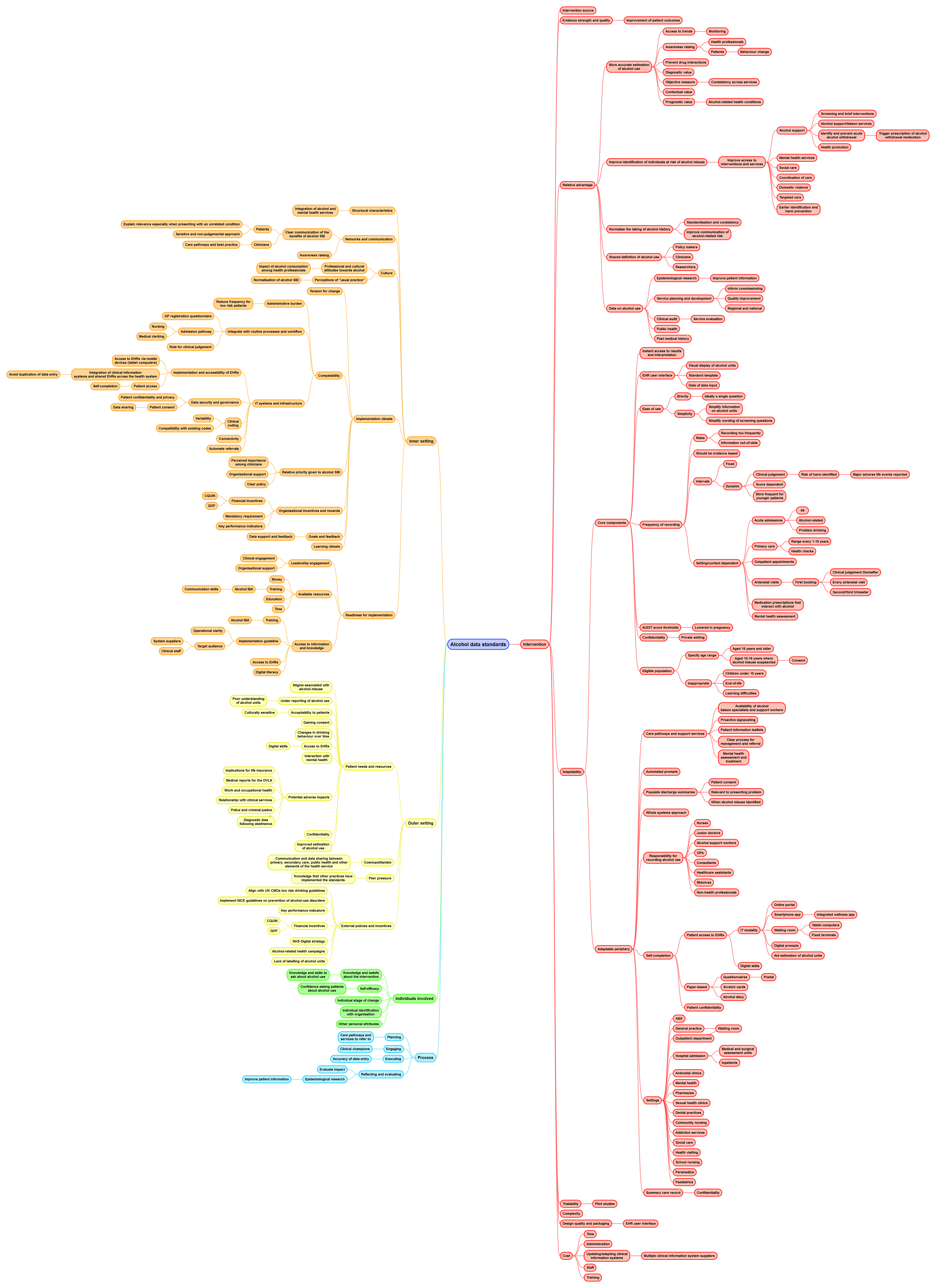

Supplement: Supplementary file 2 — This is a mind map of the key themes identified in our consultation mapped against the domains of the Consolidated Framework for Implementation Research (CFIR). (PNG 1468 kb) [file 12911_2018_612_MOESM2_ESM.png]
